# The fall of the genome protectors triad: PBRM1, SETD2, and BAP1’s impact on metabolism and immunity in clear cell renal cell carcinoma

**DOI:** 10.3389/fcell.2025.1713830

**Published:** 2026-01-12

**Authors:** Mathieu Johnson, Sandra Turcotte

**Affiliations:** 1 Atlantic Cancer Research Institute, Moncton, NB, Canada; 2 Department of Chemistry and Biochemistry, Université de Moncton, Moncton, NB, Canada

**Keywords:** ccRCC, PBRM1, SETD2, BAP1, VHL, metabolism, tumor microenvironment

## Abstract

The loss of chromosome 3p and the inactivation of the tumor suppressor gene von Hippel-Lindau (*VHL*) were identified in clear cell renal cell carcinomas (ccRCC) over three decades ago. Since then, mutations in genes for the three chromatin modulators, polybromo 1 (PBRM1), SET domain-containing 2 (SETD2), and BRCA1-associated protein-1 (BAP1), have been recognized as common in ccRCC. Although these genomic alterations are central to understanding ccRCC’s development, other deregulated cellular processes are also prominent in these tumors. Metabolic reprogramming is a key hallmark of this disease, characterized by various changes linked to the stabilization of hypoxia-inducible factors (HIF), including increased aerobic glycolysis, elevated lipid levels, and glutamine dependence for cell survival. Additionally, HIF-α stabilization plays a crucial role in regulating the immune system, thereby enhancing CD8^+^ T lymphocyte cytotoxicity. Immune checkpoint inhibitors (ICI) are now used as first-line treatments to target the often highly infiltrated tumor microenvironment of ccRCC. However, the effectiveness of ICI varies and is difficult to predict. Although emerging studies are beginning to provide insight, evidence suggests roles for PBRM1, SETD2, and BAP1 in metabolic regulation and in shaping the tumor immune microenvironment in ccRCC. Here, we review recent advances in this field and examine their impact on the management of ccRCC.

## 1 Introduction

Clear cell renal cell carcinomas (ccRCC) are the most diagnosed type of kidney cancer, representing 80% of all cases (Yo et al., 2024). Loss of heterozygosity on chromosome 3p, along with inactivation of the last copy of the tumor suppressor gene von Hippel-Lindau (*VHL*), located on chromosome 3p25-26, are key features of ccRCC (Kovacs et al., 1988; Kovacs et al., 1987; Latif et al., 1993). Aside from *VHL*, the most frequently mutated genes in ccRCC are polybromo 1 (*PBRM1*), SET domain containing 2 (*SETD2*), and BRCA1-associated protein-1 (*BAP1*), all of which are chromatin modulators found on the short arm of chromosome 3p (Dalgliesh et al., 2010; Peña-Llopis et al., 2012; Varela et al., 2011). Precisely, PBRM1 is part of the PBAF complex implicated in chromatin remodeling for DNA accessibility, SETD2 is a lysine methyltransferase best known for its role in trimethylating histone H3 on lysine 36 (H3K36me3) and regulating transcription, and BAP1 is the core component of the Polycomb repressive deubiquitinase complex (PR-DUB), which challenge the ubiquitinase activity of the Polycomb Repressive Complex 1 (PRC1) on histone H2A (H2AK119ub). Their loss is associated with genomic instability, often due to disruption in DNA damage repair and chromatin maintenance. According to the TRACERx Renal project, mutational profiles are key to ccRCC development and variability, with the inactivation of specific genes associated with cancer aggressiveness (Mitchell et al., 2018; Turajlic et al., 2018a; Turajlic et al., 2018b). Historically known to be resistant to chemotherapy and radiotherapy, patients with metastatic ccRCC have benefited from the development of targeted therapies and immune checkpoint inhibitors (ICI). However, some tumors exhibit or ultimately develop resistance to these treatments, raising the questions: why? and how? Countless studies have focused on understanding the unique features or mechanisms that could explain differences in treatment response. From these mechanisms, we observe an increase in lactic acid in the tumor microenvironment (TME) caused by the notorious Warburg effect. This has been linked to the neutralization of immune cells’ antitumoral activities and to an increase in tryptophan catabolism, leading to higher levels of kynurenine and immunosuppression (Wettersten et al., 2017; Sh et al., 2024). In this minireview, we aim to shed light on the roles of PBRM1, SETD2, and BAP1 in the tumor metabolic and immune microenvironments, and discuss how these functions could benefit the management of advanced ccRCC.

## 2 Metabolic landscape: ripples in clear water

**FIGURE 1:**
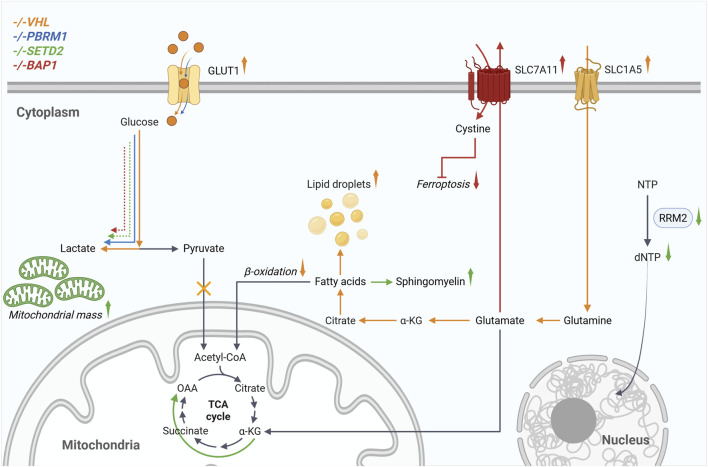
Loss of *VHL* and the associated stabilization of HIF cause the metabolic reprogramming known as the Warburg effect. This includes increased glucose uptake, glycolysis, and lactate production, which are further enhanced by loss of *PBRM1*, *SETD2*, and *BAP1*. ccRCC cells become dependent on glutamine for survival. Transmembrane transporters such as SLC1A5 enable cells to increase glutamine uptake, which is then metabolized via glutaminolysis and the reductive TCA cycle for de novo fatty acid synthesis. Additionally, β-oxidation is reduced, and lipid droplets are formed. When *SETD2* is inactivated, cells exhibit increased mitochondrial mass and oxidative phosphorylation. Downregulation of RRM2 reduces dNTP levels, which are required for DNA replication. The increased expression of SLC7A11, resulting from *BAP1* loss, enables cystine import, which subsequently leads to glutathione synthesis and resistance to ferroptosis. Created in BioRender. Johnson, M. (2025) https://BioRender.com/eg77e9h..

Kidney cancers are considered metabolic diseases caused by genetic alterations disrupting cell metabolism. This is clearly illustrated in ccRCC by the constitutive stabilization of hypoxia-inducible factors (HIFs) linked to VHL loss, leading to metabolic reprogramming. This includes increased aerobic glycolysis, glutamine dependency for fatty acid synthesis, and cytosolic accumulation of lipid and glycogen, which gives the cells a clear morphology (Rezende et al., 1999). Although many metabolic characteristics observed in ccRCC stem from VHL loss, new evidence indicates that loss of *PBRM1*, *SETD2*, and *BAP1* can also disrupt these pathways (Figure 1). While studying how the loss of *PBRM1* impacts ccRCC tumorigenesis in mice, Nargund et al. demonstrated that kidney-specific inactivation of *Vhl* or *Pbrm1* alone did not lead to tumor formation (Nargund et al., 2017). It was only when both genes were inactivated simultaneously that the mouse developed ccRCC. Additionally, RNA sequencing data identified pathways that could explain how *Pbrm1* loss contributes to tumor formation in a *Vhl*-deficient model. These results showed that both the HIF and JAK/STAT pathways, especially STAT3, were upregulated. They also revealed a decrease in the expression of genes related to oxidative phosphorylation, which occurs before activation of the mTORC1 pathway. While RNA sequencing studies have identified multiple metabolic pathways associated with variation in PBRM1 expression, only a limited number of published articles have investigated the specific role of PBRM1 in metabolism (Nargund et al., 2017; Chowdhury et al., 2016). In one of the few studies, it was shown that reintroducing *PBRM1* into Caki-2 cells decreased glucose uptake and the AKT pathway (Chowdhury et al., 2016). This was further supported by an independent study reporting results from 786-0 and SN12C cells expressing *PBRM1*-knockdown achieved through shRNA (Tang et al., 2022). Given that PBRM1 directly modulates ccRCC metabolism through HIF-1α, this prompts the question of whether SETD2 or BAP1 might also display similar HIF-1α-dependent functions. In a sepsis-induced setting, decreased SETD2 expression leads to increased HIF-1α and glycolysis, promoting M1 macrophage polarization (Meng et al., 2023). A decrease in nuclear HIF-1α signal was observed in mesothelioma cases with germline *BAP1* mutations, particularly those with biallelic mutations, which revealed BAP1’s interaction with HIF-1α (Bononi et al., 2023). In fact, Bononi et al. demonstrated that BAP1 can directly interact with and deubiquitylate HIF-1α under hypoxic conditions, thereby stabilizing HIF-1α, which was linked to increased aggressiveness in *BAP1* wild-type mesotheliomas. Evidently, these findings were examined in pathological contexts beyond ccRCC. Because HIF-1α becomes stabilized and abundant as a result of VHL inactivation in ccRCC, further detailed analysis is required to evaluate how the loss of SETD2 or BAP1 influences HIF-1α expression and the associated metabolic regulation in ccRCC.

Cells from ccRCC are well known to rely on glutamine for survival. The glutamine undergoes glutaminolysis before entering the reductive tricarboxylic acid (TCA) cycle to produce citrate for fatty acid synthesis (Metallo et al., 2012). Conversely, loss of *SETD2* was shown to enhance oxidative phosphorylation instead of the reductive TCA cycle. Knockout of *SETD2* in the 786-0 ccRCC cell line results in increased expression of peroxisome proliferator-activated receptor γ coactivator 1α (PGC-1α) and higher mitochondrial mass, leading to the rerouting of glutamine metabolism (Liu et al., 2019). Similarly, in non-small cell lung cancer, *Setd2*-inactivation led to an increase in oxidative respiration (W et al., 2023). Changes in mitochondrial morphology were also observed, with an increase in cristae within mitochondria when Setd2 was removed, although mitochondrial mass decreased. PGC-1α can also be regulated by BAP1, which directly binds to O-GlcNAcylated PGC-1α to prevent its degradation (Ruan et al., 2012). This was demonstrated in the context of insulin resistance in diabetes, where the O-GlcNAc transferase (OGT) and host cell factor-1 (HCF-1) interact with PGC-1α to promote its O-GlcNAcylation, facilitating BAP1 recruitment for deubiquitination and protecting PGC-1α from degradation. Stabilization of PGC-1α and increased hepatic gluconeogenesis are associated with diabetes, and knocking down OGT and HCF-1 in the liver of diabetic mice helped reduce gluconeogenesis and regulate glucose homeostasis. Metabolic regulations by BAP1 were also reported *in vivo* using the neutron-encoded (NeuCode) amino acid labeling method, which observed hypoglycemia and metabolic alterations in various tissues of Bap1-knockout mice (Baughman et al., 2016). Before uncovering BAP1’s role in regulating HIF-1α, the same group demonstrated how aerobic glycolysis increases with germline *BAP1* mutations (Bononi et al., 2017). Metabolic profiling was able to distinguish between individuals with wild-type *BAP1* from those with mutated *BAP1*. People with BAP1 syndrome have loss-of-function mutations in BAP1 in their primary cells, resulting in increased glucose consumption and lactate secretion. Mitochondrial respiration also declined, as shown by reduced oxygen consumption rates. These changes could not be linked to significant transcriptional differences, and no variations were observed in mitochondrial morphology, quantity, or membrane potential. Alternatively, it was demonstrated that BAP1 can be recruited to the promoter of cytochrome c oxidase subunit 7C (*COX7C*) and act as a coactivator for its transcription (Yu et al., 2010). This role was found to depend on BAP1 interaction with HCF-1 and Yin Yang 1 (YY1). However, the reduction in *COX7C*’s impact on overall cell metabolism was not further explored.

Some metabolic alterations associated with PBRM1, SETD2 and BAP1 have been shown to play important roles in the evolution of ccRCC. As previously mentioned, downregulation of oxidative phosphorylation was observed during tumor growth following *Vhl* and *Pbrm1* inactivation, which triggered mTORC1 activation (Nargund et al., 2017). In a polycystic kidney disease (PKD) model where c-MYC is overexpressed, *Setd2*-inactivation leads to an increase in sphingomyelin and the development of ccRCC tumors (Rao et al., 2023). The significance of this metabolic change was demonstrated by treating with myriocin to inhibit sphingomyelin biosynthesis, which limited tumor growth. Sphingomyelin levels were also higher in samples from patients with ccRCC who had low *SETD2* expression. With the characterization of ferroptosis over the last 2 decades, understanding its mechanism led to the discovery of BAP1’s regulation of SLC7A11. *BAP1* loss-of-function leads to increased SLC7A11 expression, facilitating enhanced cystine import, glutathione synthesis, and resistance to ferroptosis (Zhang et al., 2018). This resistance would enable *BAP1*-deficient cells to evade cell death and consequently promote tumor growth. Furthermore, some metabolic alterations can be exploited as therapeutic targets. Since SETD2 positively regulates ribonucleotide reductase M2 (RRM2), inactivating *SETD2* reduces the dNTP pool, which confers vulnerability to WEE1 inhibitors (Pfister et al., 2015).

## 3 Tumor immune microenvironment: how to tame wildfires?

The emergence of the ICI as a treatment option for cancers has radically transformed the management of patients with advanced ccRCC over the past decade. Targeted therapies, such as VEGF and mTOR inhibitors, are now mainly used in combination with ICI as first-line or second-line treatments (Kimryn Rathmell et al., 2022). ccRCC tumors are considered « hot», meaning they are inflamed with a high number of immune cells. Despite being highly infiltrated, ccRCC can still evade immune destruction. In fact, CD8^+^ cytotoxic T cells in the ccRCC TME are often exhausted, impairing their function. Notably, cancers at higher stages display a greater presence of exhausted CD8^+^ T cells and M2 tumor-associated macrophages (Braun et al., 2021). How the specific mutations found in ccRCC influence the TME is still unclear. Recently, Jiao et al. demonstrated that loss of *VHL* improves the effectiveness of anti-programmed death 1 (PD-1) therapy in a T cell-dependent context manner through the cGAS-STING pathway (Jiao et al., 2024). However, since the relationship between VHL status and the TME remains in its early stages, it is not surprising that much remains to be explored regarding PBRM1, SETD2, and BAP1 in relation to the ccRCC TME.

The loss of *PBRM1* function in melanoma was first associated with higher vulnerability to T-cell-mediated killing (Pan et al., 1979). In a genome-wide CRISPR-Cas9 screen, sgRNAs for the three unique subunits of the PBAF complex, *PBRM1*, *ARID2* and *BRD7*, were depleted after exposure to T-cells, indicating increased cell vulnerability to T-cell-mediated killing when these proteins are altered. Furthermore, tumors from *Pbrm1*-deficient B16-F10 cells exhibited increased levels of dendritic cells, a higher ratio of M1 to M2 macrophages, and enhanced activation of immune responses, particularly through the interferon-gamma (IFNγ) pathway. Using whole exome sequencing, Miao et al. showed a correlation between patients with metastatic ccRCC harboring *PBRM1* loss-of-function mutations and improved outcomes following treatments with nivolumab (Miao et al., 2018). Not long after, results from the phase 2 IMmotion150 study failed to replicate those findings, reporting no significant difference in clinical outcomes between patients with or without *PBRM1* mutations when treated with atezolizumab alone or in combination with bevacizumab (McDermott et al., 2018). Notably, patients with *PBRM1* mutations demonstrated a better response to sunitinib (McDermott et al., 2018). Braun et al. contributed to these findings by reporting improved responses to nivolumab in patients with a mutated PBRM1 and subsequently found that tumors with CD8^+^ T-infiltrated cells had a lower frequency of PBRM1 mutation (Braun et al., 2019; Braun et al., 2020). Then, by using shRNA targeting Pbrm1 in kidney Renca cells, another group demonstrated that low Pbrm1 expression correlates with reduced infiltration of both CD4^+^ and CD8^+^ T cells (Aili et al., 2021). Meanwhile, conflicting results have been reported regarding the impact of PBRM1 loss on ICI response. *PBRM1* inactivation in ccRCC cells reduces IFNγ-JAK2-STAT1 signaling, leading to reduced T cells infiltration in the TME (Liu et al., 2020). However, when assessing how these results impact the response to anti-PD-1 treatments, administering a PD-1 antibody did not affect tumor growth or the survival of mice injected with Pbrm1-knockout Renca cells (Liu et al., 2020). It is important to note that tumors from wild-type Renca cells were larger and worsened the survival of the mice compared to the *Pbrm1*-knockout. Nonetheless, using data from three different patient cohorts (TCGA-KIRK, IMmotion150, and International Cancer Genome Consortium), they showed reduced expression of multiple immune-related genes, lower immune cell infiltration, and a decreased response rate to atezolizumab alone or in combination with bevacizumab in patients with mutated *PBRM1*. Paradoxically, the exclusion of *PBRM1*’s exon 27 by alternative splicing, regulated by the splicing factor RBFOX2, altered the recruitment of the PBAF complex to *PD-L1* promoter and reduced its expression (Cho et al., 2024). In ccRCC, exon 27 was significantly excluded compared with normal tissue and was associated with increased RBFOX2 expression. While patients with *PBRM1* mutations showed better overall response, high RBFOX2 expression improved survival in those with *PBRM1 wild-type* when treated with nivolumab. Altogether, there appears to be a consensus that *PBRM1*-mutated ccRCCs are associated with a less inflamed TME; however, the specifics of how these tumors respond to immunotherapy remain a subject of debate.

The role of SETD2 in the immune response was first reported in hepatitis B virus infection (Chen et al., 2017). Chen et al. demonstrated that SETD2 directly interacts with STAT1, methylating STAT1 on lysine 525 to promote its phosphorylation and activation. Additionally, the cGAS-STING pathway, which is closely connected to the activation of type I IFN and STAT1 pathways, was shown to play a significant role in the vulnerability of *SETD2*-deficient ccRCC to ATR inhibition (Liu et al., 2023). Alternatively, STAT1 was reported to be negatively regulated by nuclear receptor subfamily 2, group F, member 1 (NR2F1), which is repressed when *SETD2* is inactivated, thereby allowing upregulation and activation of STAT1 (Zheng et al., 2023). With SETD2’s essential role in DNA repair, its inactivation has been associated with an increased tumor mutational burden, which can enhance T cell recognition via the major histocompatibility complex class I (MHC-1). Additionally, they validated these results using TCGA data in a ccRCC cohort, where NR2F1 expression was decreased when SETD2 was either absent or expressed at low levels. Although there has been increased interest in studying SETD2 in ccRCC over the past few years, research on SETD2 and its role in influencing the tumor immune microenvironment of this cancer has barely begun. A handful of studies have been performed in the context of pancreatic cancer and suggested that *SETD2* loss modulates the TME in an immunosuppressive state (Niu et al., 2024; Niu et al., 2023). *Setd2* inactivation was associated with increased neutrophil infiltration and the suppression of cytotoxic CD8^+^ T cells (Niu et al., 2023). Mechanistically, a decrease in H3K36me3 caused by the loss of *Setd2* was followed by an increase in H3K27me3. This resulted in downregulation of the *Cxadr* cell adhesion molecule, thereby enhancing AKT pathway activation and increasing expression of C-X-C motif chemokine ligand 1 (*CXCL1*) and granulocyte-macrophage colony-stimulating factor (*GM-CSF*), facilitating the recruitment of neutrophils with higher levels of PD-L1. In a pan-cancer analysis including the TCGA-KIRC cohort, data showed an overall correlation between *SETD2* deficiency, a more prominent immune activation signature, and higher tumor mutational burden (Lu et al., 2021).

As for *BAP1*, its loss has recurrently been associated with a more inflamed TME. Following analysis of two clusters of ccRCC patients, identified as the inflamed and non-inflamed subtypes, *BAP1* mutations were found to be significantly more common in the inflamed subtype (Wang et al., 2018). In this study, patients with *PBRM1* mutations were found to be more heterogeneous in both clusters, which may explain the conflicting results presented earlier. The inflammatory effect of *BAP1* loss in the TME is also noticeable in malignant pleural mesothelioma, a cancer frequently mutated in *BAP1* (Xu et al., 2024). Similar to PBRM1 and SETD2, it has been suggested that BAP1 also regulates the interferon pathways. Specifically, BAP1 typically promotes the activation of IFNβ and STING mediated by HIF-2α, which helps inhibit tumor growth through the activation of interferon stimulated gene factor 3 (ISGF3) (Langbein et al., 2022). Activation of this pathway was sufficient to limit tumor growth of cell line-derived xenografts from *BAP1*-inactivated Ren-02 cells. As an alternative therapeutic approach for metastatic ccRCC, preliminary data have shown a better response with a cell line-derived xenograft with a *BAP1*-mutated ccRCC cell line, 769-P, when treated with the oncolytic vaccinia virus (JX-594) (Park et al., 2025). Further experiments are needed to confirm whether sensitivity to JX-594 is truly linked to BAP1 mutations rather than to other genetic changes in the 769-P cell line. Additionally, although it is not the primary focus of this mini-review, it is worth noting that multiple studies have shown roles for these three genes in the development and regulation of various immune cells (Meng et al., 2023; Zhang et al., 2024; Chu et al., 2020; Ding et al., 2022; Takagi-Kimura et al., 2022; Ji et al., 2019; Chen et al., 2024; Li BE. et al., 2023).

## 4 At the crossroad of metabolic alterations and tumor immune microenvironment modulation

The characterization of the impacts associated to the loss of these three commonly mutated genes offers new opportunities to manage advanced ccRCC, such as using them as biomarkers for treatment prediction, thereby assisting in selecting the most suitable treatment for each patient, aligning with precision medicine. With their specific roles in regulating metabolic and immune pathways that have only recently begun to be understood, mechanistic studies often overlook how these genes are interconnected and how the loss of one can impact the functions of others. Alternatively, many studies on BAP1 or SETD2 have been conducted in other cancers and need to be confirmed in ccRCC.

Classification of ccRCC tumors based on their immune or metabolic signatures provides crucial insights into tumor progression and treatment options. In 2019, Clark et al. used proteogenomic analysis to classify ccRCC into four immune subtypes: CD8^+^ inflamed, CD8- inflamed, VEGF immune-desert, and metabolic immune-desert (Clark et al., 2019). *PBRM1* mutations were more common in the VEGF immune-desert subtype, which was associated with lower-grade tumors and increased expression of proteins involved in glycolysis, hypoxia, and the mTOR pathway. Conversely, *BAP1* mutations were more prevalent in the CD8^+^ inflamed subtypes, which were associated with higher-grade tumors and higher protein expression in pathways such as OXPHOS, fatty acid metabolism, and the adaptive immune response. A subsequent multi-omics study classified ccRCC into tumor immune subtypes (IM1-4) similar to those identified previously (Hu et al., 2024). This study characterized subtypes by their endothelial, stromal, and immune cell signatures. Both IM1 and IM2 signatures indicate high endothelial cell levels, with IM1 showing stromal cell enrichment and immune exclusion, while IM2 shows depletion of stromal and immune cells. IM3 displays low levels of endothelial and stromal cells, but infiltration by T cells and tumor-associated macrophages (TAMs). IM4 features high levels of stromal cells and TAM, along with intermediate levels of T cells. They also stratified tumors based on metabolic signatures, revealing a group with fewer lipid droplets, predominantly in IM4. Their extended data clearly show that *PBRM1* mutations are present across all four subtypes, whereas *SETD2* and *BAP1* mutations are absent in IM1 but more common in IM3 and IM4.

Preliminary results from the TRACERx Renal project were first published nearly a decade ago; however, evolutionary subtypes are still underrepresented in research, especially in studies using simpler models such as 2D cell cultures (Turajlic et al., 2018b). The development of new experimental models, such as spheroids, organoids, patient-derived xenografts, and genetically engineered mouse models, offers opportunities, despite limitations, to study how cancer cells interact with the immune system. Nevertheless, in the most recent publication from the TRACERx Renal project, they investigated the relationship between prominent genomic alterations observed in the seven evolutionary subtypes and the tumor immune microenvironment (Fernández-Sanromán et al., 2025). By comparing paired samples from the primary tumor and the metastases, the loss of *SETD2* was associated with a transition to an immunosuppressive TME. The loss of chromosome 9p was the only other somatic alteration showing this tendency. Among the three genes, the loss of *SETD2* is typically a subclonal event, whereas the loss of *PBRM1* or *BAP1* usually occurs as a clonal event earlier in tumor progression. It is also unclear how SETD2 impacts the aggressiveness of the cancer. However, with increasing interest in that gene, new evidence, such as the one reported from the TRACERx Renal project, highlights SETD2 as a key player and a potential therapeutic target. We recently reported that *SETD2* inactivation in ccRCC expressing wild-type *VHL* sensitizes cells to STF-62247 resulting in a cell cycle block in S-phase (Johnson and Turcotte, 2025). As Liu et al. stated and further demonstrated in their study, targeting the S-phase DNA damage repair network can improve the response to immune checkpoint inhibitors (Liu et al., 2023). According to this theory, co-treatment with STF-62247 and ICI could produce a synergic cytotoxicity. Other proteins and pathways related to metabolism have also been suggested as potential therapeutic targets for cancer cells lacking *SETD2*, including the PI3K pathway, WEE1, and the mTORC1 signaling pathway (W et al., 2023; Pfister et al., 2015; Terzo et al., 2019).

Overall, PBRM1, SETD2, and BAP1 have been associated with specific metabolic and immune alterations; however, this area remains underexplored and holds promise. Targeting metabolic deregulation associated with these mutated genes offers a promising strategy for developing new therapies, especially when combined with immunotherapy. The increase in lactate secretion related to aerobic glycolysis, which has been shown to be amplified by the loss of PBRM1 under hypoxic conditions, could be targeted with lactate-focused treatments such as the monocarboxylate transporter 1 (MCT1) inhibitor AZD3965 (Tang et al., 2022; Halford et al., 2023). However, aside from one study showing a positive response to AZD3965 treatment in xenograft models, specifically when lysine acetyltransferase 2A (KAT2A) was overexpressed, and MCT1 was upregulated, no clinical data demonstrate the response of this treatment in patients with ccRCC (Li X. et al., 2023; Guo et al., 2021). Nonetheless, there is still a long road ahead and more studies needed on metabolic changes caused by the loss of these genes and their influence on the tumor immune microenvironment before this knowledge can be fully utilized to guide clinical decisions in ccRCC.
